# Long-Range Periodic Patterns in Microbial Genomes Indicate Significant Multi-Scale Chromosomal Organization

**DOI:** 10.1371/journal.pcbi.0020002

**Published:** 2006-01-13

**Authors:** Timothy E Allen, Nathan D Price, Andrew R Joyce, Bernhard Ø Palsson

**Affiliations:** 1 Department of Bioengineering, University of California San Diego, La Jolla, California, United States of America; 2 Bioinformatics Program, University of California San Diego, La Jolla, California, United States of America; University of California San Diego, United States of America

## Abstract

Genome organization can be studied through analysis of chromosome position-dependent patterns in sequence-derived parameters. A comprehensive analysis of such patterns in prokaryotic sequences and genome-scale functional data has yet to be performed. We detected spatial patterns in sequence-derived parameters for 163 chromosomes occurring in 135 bacterial and 16 archaeal organisms using wavelet analysis. Pattern strength was found to correlate with organism-specific features such as genome size, overall GC content, and the occurrence of known motility and chromosomal binding proteins. Given additional functional data for Escherichia coli, we found significant correlations among chromosome position dependent patterns in numerous properties, some of which are consistent with previously experimentally identified chromosome macrodomains. These results demonstrate that the large-scale organization of most sequenced genomes is significantly nonrandom, and, moreover, that this organization is likely linked to genome size, nucleotide composition, and information transfer processes. Constraints on genome evolution and design are thus not solely dependent upon information content, but also upon an intricate multi-parameter, multi-length-scale organization of the chromosome.

## Synopsis

For more than a decade, the genetic material for a growing number of microbial organisms has been determined experimentally using genome sequencing techniques. These sequenced genomes provide researchers with an abundance of information regarding the composition and capabilities of each organism since they serve as “parts lists” that specify the protein machinery that each cell generates. However, genomes are not merely “lists” but also are typically arranged in nonrandom order. It is thought that this order may be related to some extent to the way in which each genome is packed into the tiny confines of a cell (often more than 1,000-fold packing). The authors have used signal processing methods to identify long-range spatial patterns in the arrangement of most sequenced microbial genomes, and they have related the degree of organization in each genome to various characteristics specific to the corresponding organisms. They have also analyzed in detail the degree of overlap among patterns in numerous different kinds of data for a model bacterial organism, *Escherichia coli*. Their results conclusively demonstrate that there are significant evolutionary constraints that act upon genome organization as well as genome content, and that the interplay between organization and function cannot be ignored in understanding fundamentally how a microbial cell works.

## Introduction

Genomes in prokaryotic organisms typically are packed tightly into a nucleoid where they carry out multiple functions simultaneously [1,2]. The condensed DNA within the bacterial nucleoid must not only be efficiently replicated and segregated during cell division [3], but it must also simultaneously participate in the information transfer processes of transcription and translation [4]. Recent studies have significantly advanced our understanding of the ultrastructural and multifunctional organization of prokaryotic chromosomes. DNA in Escherichia coli has been found to be packed into supercoiled domains ranging 2–66 kb and averaging ~10 kb [5]. At a slightly longer length-scale, studies using fluorescence in situ hybridization have revealed that the origin and terminus of replication in E. coli gravitate toward the poles of the cell throughout replication, but both migrate to the mid-cell region just prior to the initiation of chromosome replication [6]. Fluorescence experiments in synchronized cultures of the aquatic bacterium Caulobacter crescentus have revealed the cellular location of 112 individual chromosomal loci throughout replication and cell division [7]. In addition to these imaging techniques, genetic dissection has been used to identify four macrodomains and two less-structured regions in the E. coli chromosome [8]. Two of these macrodomains were consistent with those found near the origin and terminus of replication using fluorescence in situ hybridization [6]. However, many issues remain unresolved regarding the intricacies of this arrangement, and particularly the relationship between chromosomal ultrastructure and the processes of transcriptional regulation and protein synthesis [4,9].

**Figure 1:**
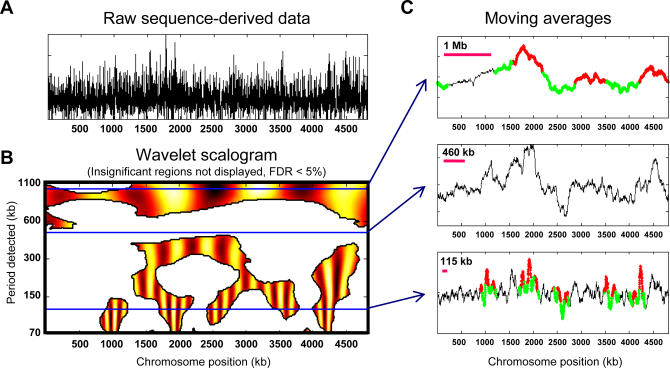
Approach for Detecting Genome Position-Dependent Patterns (A) Raw sequence-derived data often contain patterns with respect to chromosome position that are not obvious from casual observance. (This example is for the fractional gene density per kilobase for *Salmonella enterica* serovar Typhi strain CT18.) (B) Wavelet analysis was used to generate a scalogram showing significant chromosome position-dependent patterns in gene density over varying periodicities. The level of significance of the patterns was determined by randomizing the order of the raw sequence data 200× and recomputing the real and imaginary portions of the Morlet wavelet transform values at each point in the scalogram for each randomization. Regions having an FDR greater than 5% are not displayed (white). The pattern strength for this dataset is 33%. (C) To facilitate the interpretation of the wavelet scalogram, three examples are shown for the moving averages of the raw data at three different length scales: 1 Mb, 460 kb, and 115 kb. Regions highlighted in red/green indicate significant regions of the scalogram at that scale that lie above/below the mean real transform value.

Several studies have revealed that genes in bacterial nucleoids tend to be arranged along the long axis of the cell (in the case of rod-shaped bacteria) so as to preserve the linear order of the genes along the chromosome [6,7,10,11]. Given this linear arrangement, prokaryotic genome sequences inherently contain useful information relating to chromosomal ultrastructure since they provide numerous properties as a function of chromosome position [12]. However, the inference of 3-D genome-packing from direct examination of the raw sequence is somewhat challenging at the short length-scales of the nucleotide, gene, or operon (1 bp–10 kb) due to the inherently one-dimensional nature of sequence data and hence the considerable sequence noise over shorter scales. Accordingly, various averaging and filtering methods have been used to identify long-range (i.e., >10-kb) position-dependent patterns in genome-associated properties [12–14]. In order to detect such long-range periodic patterns in inherently noisy chromosome position-dependent data, wavelet analysis has been used in several studies [13,15] (Figure 1). This method has previously been used to detect patterns in gene orientation [14], DNA-bending profiles [16], and gene expression data [17,18] in prokaryotes, as well as GC/AT skew oscillations in human chromosomes [19]. These studies have revealed that genome sequences are generally nonrandom with respect to chromosome position, and that long-range correlations in certain properties (e.g., gene orientation; [14]) exist across many different length-scales.

As more prokaryotic genome sequences become available, it should be increasingly possible to relate the quantitative degree of genome organization to global properties of each organism, including the presence of known nucleoid-binding proteins [20], organism taxa, and genome size and composition. Observed correlations may indicate constraints that affect (or are affected by) genome organization. Furthermore, a study of genome position-dependent patterns in heterogeneous data types in a well-studied model organism such as E. coli (e.g., gene expression versus specific codon preferences) may reveal properties that are spatially linked. Therefore, the need exists to define an unbiased, quantitative measure of genome organization from sequence-derived data, compute this quantity for numerous sequenced prokaryotic genomes, relate this quantity to global properties of each organism, and determine the spatial coupling of multiple heterogeneous properties for a well-studied model organism.

In this study, we address these needs by employing wavelet analysis in concert with a bootstrap significance test (Materials and Methods) to compute the pattern strengths of chromosome position-associated datasets derived from 163 sequenced prokaryotic chromosomes. This pattern strength provides a measure of the nonrandom nature of sequence-derived data that is independent of genome length. We then computed the pattern strength of genome position-dependent properties for nearly every sequenced prokaryotic genome, and we related this measure to taxonomic and physiological characteristics of each organism. Finally, we examined disparate genome position-dependent data available for E. coli to determine properties that are spatially correlated over multiple length-scales. Our results demonstrate that the degree of organization in bacterial genomes is highly variable and correlates with specific properties, and our analysis of patterns in multiple E. coli datasets supports the notion that the overall organization of the bacterial chromosome results from the simultaneous optimization of functional and structural constraints.

## Results/Discussion

### Pattern Strengths of Sequenced Prokaryotic Organisms

**Figure 2:**
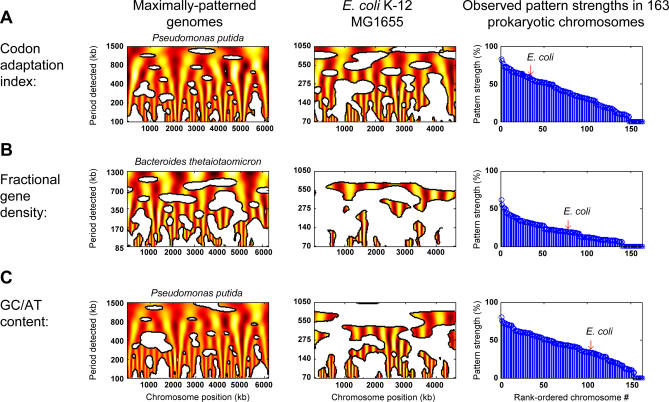
Generality of Chromosome Position-Dependent Patterns in Sequence Properties for 163 Prokaryotic Chromosomes Continuous wavelet scalograms were computed for most prokaryotic chromosomes sequenced through January 2005 to identify patterns in CAI per gene (A), fractional gene density per kilobase (B), and GC/AT content per kilobase (C). The colored portions of the scalogram indicate significant periodic patterns (FDR < 5%). The degree of patterning for each prokaryotic sequence and each parameter (called the fractional pattern strength) was taken as the percentage of the area of the scalogram containing significant patterns. The first column shows the scalograms for the maximally patterned chromosome found for each sequence property. For reference, the second column shows these scalograms for *E. coli* K-12 MG1655. The third column shows the rank-ordered fractional pattern strengths for the 163 sequenced prokaryotic chromosomes that were analyzed, with *E. coli* indicated relative to the other chromosomes on each plot.

Using the pattern detection method described (Materials and Methods), we computed the pattern strengths for the GC/AT content, fractional gene density, and codon adaptation index (CAI) derived from 163 sequenced prokaryotic chromosomes (Figure 2). The average pattern strength for GC/AT content was 40% (standard deviation [SD] = 20%), 19% (SD = 14%) for gene density, and 37% (SD = 22%) for CAI. (The descriptive statistics for these distributions are summarized in Table 1.) The high SDs indicate that significant chromosome position-dependent patterns vary extensively for different organisms. The relative lack of patterning in gene density is a result of the low positional variability due to the short intergenic regions found in the generally gene-dense prokaryotic organisms. Rank-ordering the genomes by pattern strength revealed the variation in the degree of patterning in sequence-derived parameters in these chromosomes (Figure 2, right column). Table 2 lists the chromosomes containing the strongest and weakest patterns for each parameter, and the scalograms corresponding to the strongest patterns are indicated in the left column of Figure 2. The scalograms for E. coli are provided for reference (Figure 2, middle column). Significant patterns were also detected in gene orientation (i.e., strand) for all but one of the chromosomes (Table S1).

**Table 1:**
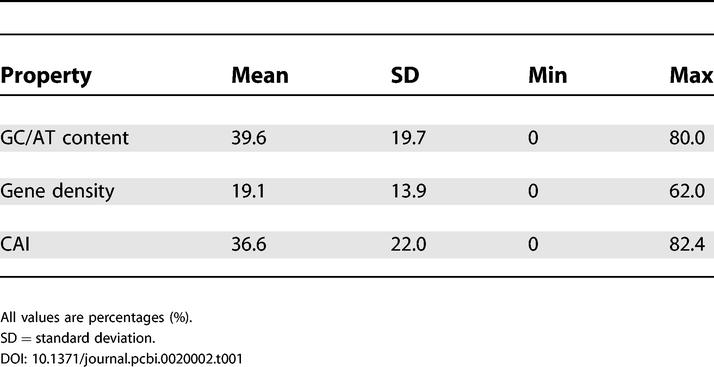
Descriptive Statistics for Pattern Strengths in GC/AT Content, Gene Density, and CAI across 163 Prokaryotic Chromosomes

**Table 2:**
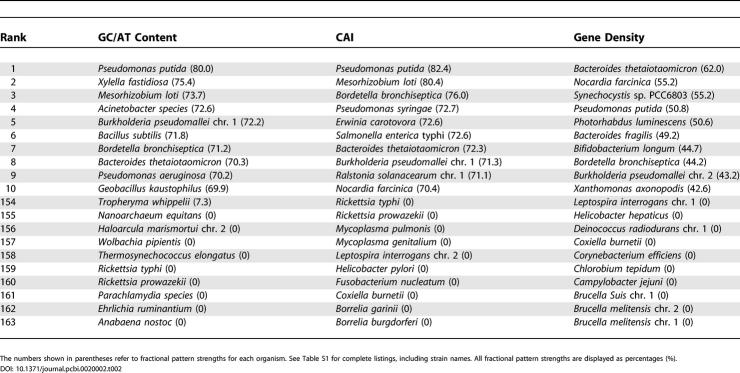
Organisms Exhibiting Either very High or very Low Chromosome Position-Dependent Patterns in Sequence-Derived Data

### Correlation of Pattern Strengths to Organism-Specific Properties

**Figure 3:**
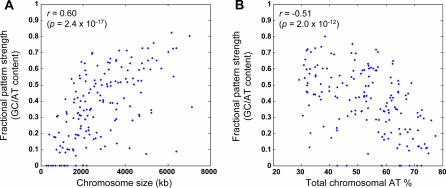
Correlations between Sequence-Derived Properties for 163 Prokaryotic Chromosomes (A) Correlation of fractional pattern strength in GC/AT content with chromosome length. (B) Anti-correlation of fractional pattern strength in GC/AT content with total chromosomal AT%. The correlation coefficients and associated *p-*values are indicated on each graph.

Pattern strengths in the sequence-derived parameters for each chromosome were compared with global properties such as genome length, total AT composition, organism taxon, and the presence of specific nucleoid-binding proteins. Pattern strengths in CAI and GC/AT content were found to be weakly but significantly correlated with genome size (*r* = 0.60, *p* = 2.4 × 10^−17^; Figure 3A) and anti-correlated with total AT composition (*r* = −0.51, *p* = 2.0 × 10^−12^; Figure 3B). These correlations are consistent with previously observed correlation between genome size and GC-content [21] and suggest an evolutionary requirement for greater genome organization in larger and more GC-rich organisms. However, a causal relationship among these three parameters is impossible to determine at this point. The potential evolutionary constraint regarding genome size may simply be the function of a requirement for a higher-level organization necessary to pack larger genomes into the bacterial cell. The tendency of GC-rich genomes to be more highly patterned is likely linked to physical constraints imposed by the more rigid DNA resulting from the triple hydrogen bond between guanine and cytosine.

We then examined correlation of pattern strength with particular organism-specific characteristics relating to taxon, gram stain, cell shape, and the presence of particular classes of proteins in each organism (summarized in Table 3). The Wilcoxon rank-sum test (*p* < 0.05) was used to assess significance. With respect to organism taxa, patterns in CAI were found to be stronger among the proteobacteria and weaker among the mollicutes and spirochetes. Cell-shape biases in pattern strength included a preference for stronger patterns in rod-shaped bacteria and weaker patterns in spiral-shaped bacteria. No other correlations relating to organism taxa, staining characteristics, or cell shape were observed. However, this analysis is inherently biased by the particular genomes that have been sequenced to date and are thus somewhat skewed toward enteric bacteria and pathogens. As the physiological and morphological diversity of sequenced prokaryotes increases, more definitive conclusions can be drawn regarding possible correlation between genome patterning and such properties as organism lifestyle and cell shape.

**Table 3:**
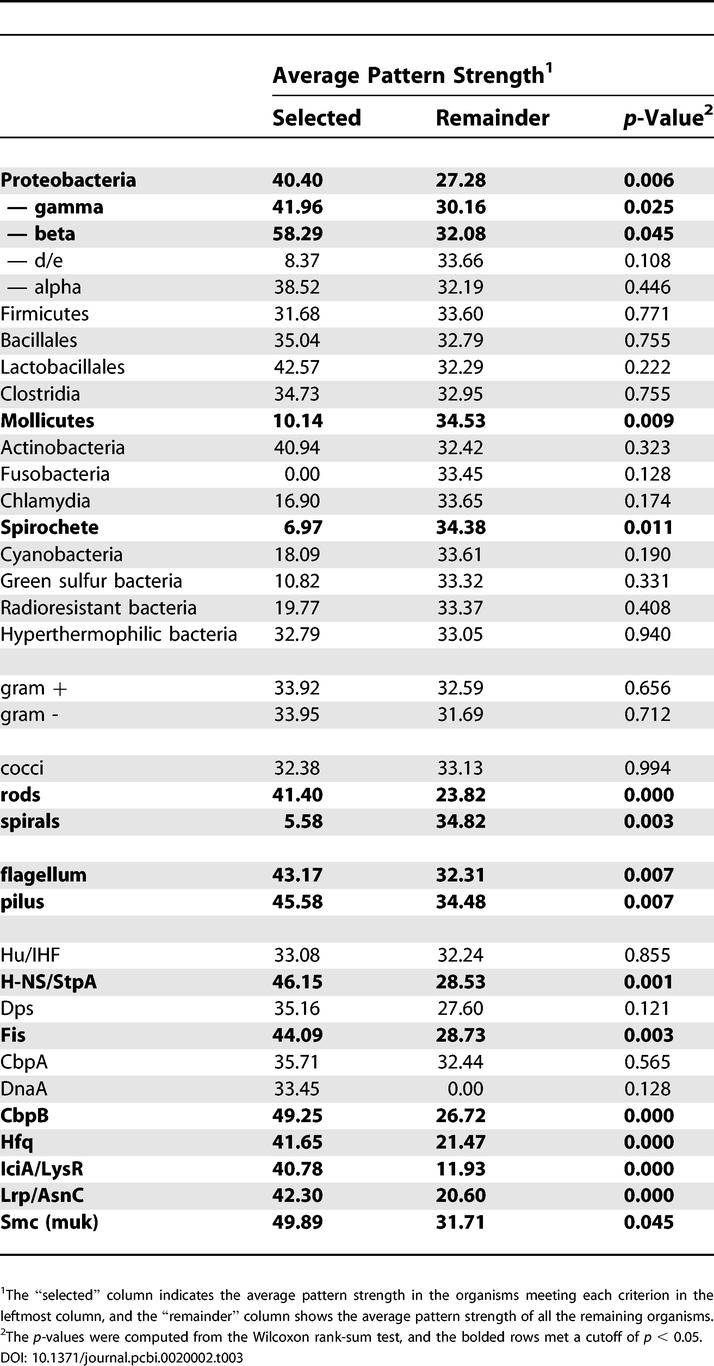
Correlation between Pattern Strength in CAI and Organism Taxon, Gram Staining, Cell Shape, and the Presence of Known Motility and Nucleoid Proteins

Genomes exhibiting the strongest patterns in CAI and GC/AT content had a higher likelihood (Wilcoxon rank-sum *p* < 0.05) of containing genes for flagella and pili than would be expected if the existence of these structures were uncorrelated with pattern strength. As shown in Table 3, the presence of genes encoding the specific nucleoid binding proteins H-NS, Fis, CbpB, Hfq, IciA, Lrp, and Muk was also found to be correlated with overall patterning in CAI. Comparisons of pattern strengths for each sequence-derived parameter revealed no significant correlations, with the exception of GC/AT content versus CAI (*r* = 0.74, *p* = 5.6 × 10^−29^). This correlation reflects the fact that CAI and GC/AT content are not actually independent properties, since GC-rich stretches of DNA will favor synonymous codons containing G and C.

### Overlap of Patterns in Heterogeneous Datasets in E. coli

**Figure 4:**
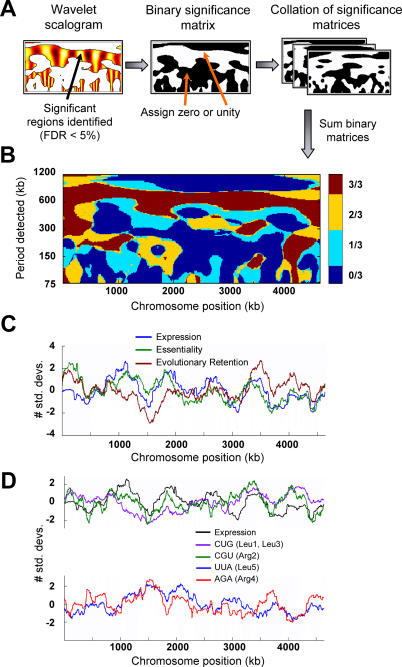
Correlation of Specific Chromosome Position-Dependent Patterns in *E. coli* Functional Properties (A) Wavelet scalograms calculated for gene expression, gene essentiality, and evolutionary retention index were converted to a binary significance matrix by setting each significant point in a scalogram (FDR < 5%) to unity and each non-significant point to zero. (B) These binary matrices were summed across the three properties listed above to determine chromosome position-dependent patterns that were consistent across the different properties, and the resulting map was color-coded according to how many of the properties shared significant patterns. The red-colored segments indicate the periods and chromosome positions at which all three properties exhibited significant patterns. The averaged data have been normalized such that the mean is zero and the tick marks indicate SDs from the mean value. (C) Correlation of gene expression, essentiality, and evolutionary retention averaged at a window of 325 kb (650-kb period). (D) Correlation of gene expression with intragenic codon preferences for two of the major codons encoding leucine (CUG) and arginine (CGU), and anti-correlation of these with preferences for the corresponding minor codons, UUA and AGA, at a moving average of 325 kb. The labels are as described above in (C).

Since a 600–650 kb periodic pattern has previously been detected in E. coli gene expression [17,18,22], the above results motivated an assessment of chromosome position-dependent patterns in functional properties specifically in E. coli (in addition to the patterns in GC/AT content, CAI, gene density, and gene orientation discussed above). Correlation of similar patterns in these heterogeneous datasets allows for an evaluation of the structural and functional organization of the E. coli genome. Binary matrices of significant pattern density regions were generated for a *p-*value cutoff corresponding to a specified false discovery rate (FDR) [23] (FDR < 5% for our analysis). Unity was assigned to regions in the scalogram deemed to have statistically significant patterning and zeros assigned elsewhere (Figure 4; Materials and Methods). For any given collection of datasets, the corresponding binary pattern-significance matrices can then be collated and visualized as a contour plot to reveal the extent of overlap in regions of the wavelet scalograms sharing significant *p-*values (Figure 4A).

**Figure 5:**
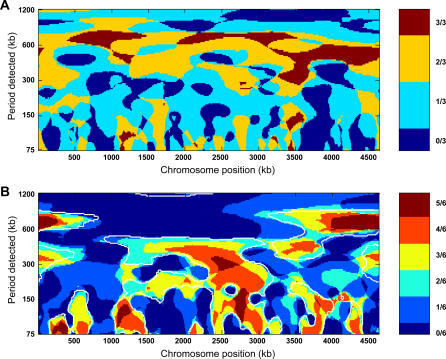
Overlay Plots of Significant Regions of Wavelet Scalograms for Various *E. coli* Parameters (A) Degree of significant pattern overlap in expression, gene density, and codon adaptation in *E. coli.* Binary matrices corresponding to significant regions of wavelet scalograms (FDR < 5%) for gene expression, CAI, and fractional gene density in *E. coli* were summed as described in Materials and Methods. A periodic pattern of 600–650 kb can be seen across nearly three-quarters of the chromosome. (B) Degree of significant pattern overlap sequence-derived DNA-bending parameters in *E. coli*. Binary matrices corresponding to significant regions of wavelet scalograms (FDR < 5%) for intrinsic curvature, DNAseI sensitivity, protein-induced deformability, propeller twist, stacking energy, and nucleosome position preference in *E. coli* [12] were summed as described in the text. The white contour lines outline the significant regions of the wavelet scalogram for GC/AT content, thus demonstrating that these parameters are not independent.

In analyzing the overlap of patterns in functional genome position-dependent data in E. coli, we observed that gene expression [17], gene essentiality [24], and the evolutionary retention index [24] contain significant periodic patterns overlapping at the 650-kb length-scale (Figure 4B) and are strongly (positively) correlated (Figure 4C). Significant patterns in gene expression at the 600–650-kb period were also found to overlap with patterns in fractional gene density and CAI over most of the genome (Figure 5A). This observation is consistent with the known coupling of transcription and translation in prokaryotes [25], since shared positional biases in CAI and expression imply that codon usage (which affects translation) is spatially coupled to gene expression (transcription). Additionally, large-scale periodic patterns (most at the ~650-kb length-scale) in the intragenic preference of specific synonymous codons were detected in E. coli, implying consequent positional biases in the corresponding tRNA species. Thus, certain tRNA species will be preferentially demanded over specific regions of the chromosome; e.g., different tRNAs for arginine and lysine will be demanded at regions of either high or low gene expression at the 600–650-kb length-scale (Figure 4D). The observed chromosome-position biases in gene expression and specific codon preferences in E. coli, along with the codon adaptation patterns observed in most of the 163 prokaryotic chromosomes analyzed in this study, suggest the existence of spatial gradients in the functional state of specific domains within each folded nucleoid [26]. These gradients may lead to spatial gradients in tRNA concentration that result from differential local demands for specific tRNA species [27].

Analysis of all 163 chromosomes revealed that long-range patterns in synonymous codon usage (CAI) are not strictly independent from those in GC/AT composition. However, patterns in sequence-derived DNA-bending parameters for E. coli (e.g., intrinsic curvature, propeller twist, stacking energy, etc.) almost completely overlap with patterns in GC/AT content (Figure 5B). As described previously, the GC/AT content reflects the average bendability of the chromosome over multiple length-scales [12]. Thus, the observed correlation of pattern strengths in CAI and GC/AT content implies a general coupling of information storage with chromosomal bending. The strongest overlap in nucleotide sequence content and sequence-derived bending parameters in E. coli consists of a 600–650-kb periodic pattern near the origin of replication between the 3,800–250-kb nucleotide coordinates (82′ to 5′). This region closely coincides with the *E. coli–*origin macrodomain detected in previous studies cited [8], and the structural regularity at the 600-kb length scale may facilitate localization of the origin to one of the cell poles during replication [6]. These DNA-bending associated datasets also contain localized periodic patterns at length scales on the order of 80–100 kb that occur in specific regions of the chromosome. The maximum pattern density in GC/AT content in this range occurred at the 74-kb period, containing eight localized patterns. Six of these eight pattern-rich segments were found to be significantly enriched (hypergeometric *p* < 0.001) with genes belonging to particular functional classes [28], which included prophage-related genes and genes encoding membrane-associated proteins (flagellar, energy production and transport, and cell-surface antigens). The enrichment of patterned regions with genes of extrachromosomal origin implies a preferred regularity in chromosome structure and nucleotide content that facilitates foreign DNA incorporation. In the case of the regions enriched in membrane-associated proteins (flagellar, cell surface, etc.), the translocation of these proteins [29] may be enhanced by regular structure at the 80–100-kb length-scale.

**Figure 6:**
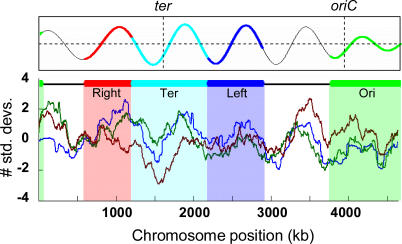
Comparison of *E. coli* Gene Expression, Essentiality, and Evolutionary Retention at 600–650-kb Length Scale with Experimentally Identified Chromosome Macrodomains [8] The four shaded regions correspond to four macrodomains identified previously based upon the frequency of recombination events following genetic dissection of the *E. coli* chromosome. The two unshaded regions correspond to less-structured macrodomains. The traces in the lower panel are exactly as described in Figure 4C. The upper panel is a section of the wavelet scalogram for *E. coli* gene expression at a 650-kb period. Segments of this wavelet transform trace have been colored to correspond to the experimentally identified chromosome macrodomains.

Genome topology has been shown to be a selection target in the long-term evolution of E. coli [30]. Our results demonstrate that prokaryotic genomes generally possess significant organization that increases with genome size, overall GC composition, and the presence of several known nucleoid-binding proteins. Thus, genome composition and size may impose additional constraints on the evolution of gene order and chromosomal arrangement in prokaryotes. Given that the spatial organization of chromosomal loci within a replicating E. coli cell is linearly ordered along the cellular axis [6,11], the analysis presented here would imply the existence of six subchromosomal functional domains in the E. coli genome [22]. This notion of highly expressed topological domains has been suggested before [31] and is consistent with the macrodomains elucidated by genetic dissection of E. coli [8]. The boundaries of those four domains and two less-structured regions [8] align with the boundaries of the regions of high and low gene expression, gene essentiality, and evolutionary retention in E. coli at the 600–650-kb length-scale (Figure 6). The observed patterns reveal that information transfer and chromosomal organization within the E. coli nucleoid are spatially interlinked.

### Implications and Conclusions

As demonstrated in the analyses described above, genome sequences and sequence-derived properties are significantly patterned (i.e., non-randomly distributed) with respect to chromosome position in most of the prokaryotic genomes sequenced to date (Figure 2). The degree of patterning in a bacterial organism is positively correlated with genome size, overall GC-content, the presence of several known nucleoid-binding proteins, and the presence of flagellar proteins (Figure 3; Table 3). These results strongly suggest the existence of structural constraints imposed by organism-specific features on the evolution of genome organization and base-pair composition in each organism.

In E. coli, a more detailed analysis of available data demonstrates that patterns in multiple disparate properties are interlinked (Figures 4 and 5). The consistency of the 650-kb chromosome macrodomains identified using wavelet analysis of expression data [17] with those identified from genetics experiments [8] indicates that large-scale genome packing is indeed linked to transcription, as has been previously hypothesized [4] (Figure 6). This work has additional implications for de novo genome design [32], in that gene order and composition—and the resulting chromosomal ultrastructure—are significant design variables that will likely need to be taken into account. Given the non-random distribution of these parameters in nearly all sequenced prokaryotes, as well as the linked nature of disparate parameters in E. coli, it is clear that any genome design endeavor will involve a multivariable, multidimensional optimization problem. The present study constitutes an early step in the evolution of systems biology from analyses of component (1-D) and systemic (2-D) annotations [33] toward the systems analysis of 3-D genome organization.

## Materials and Methods

### Chromosome position-associated datasets.

Datasets were analyzed from most prokaryotic genome sequences published through January 2005 and were downloaded from the CBS Genome Atlas Database [34] (http://www.cbs.dtu.dk/services/GenomeAtlas). Four types of chromosome position-dependent data were analyzed for 151 prokaryotic organisms (corresponding to 163 chromosomes in 16 archaeal and 135 bacterial organisms): 1) GC/AT content averaged in kilobase bins, 2) gene orientation (i.e., strand), 3) fractional gene density (defined as the number of genes—or fractions of genes—per kilobase), and 4) CAI [35] per gene. For the CAI, we used the global codon usage as the reference set to maintain consistency, since the highly expressed genes for some of the organisms may not be predictable a priori. GC and AT content are by definition inversions of one another and are strictly anti-correlated, so any patterns present in either property will be identical. Thus, patterns in these properties are simply referred to as patterns in GC/AT content. The analysis of additional data from E. coli K-12 MG1655 included sequence-derived biophysical parameters averaged across 1-kb segments [12], gene classifications and product locations [28], gene expression [17], gene essentiality [24], and evolutionary retention indices computed based upon homology with 32 representative bacterial sequences [24].

### Pattern detection by wavelet analysis and significance testing.

Wavelet analysis, reviewed in detail elsewhere [36], is an approach whereby irregular patterns in biological data may be elucidated [14,15,17–19,37]. In short, each genome-scale dataset was ordered according to position along the chromosome. These ordered data, *f*(*x*) (where *x* is defined as the nucleotide position along the chromosome), were then continuously integrated using a family of filter functions to obtain a transform value for numerous filter widths (i.e., scales, designated *a*) centered at each position *x* in the dataset:


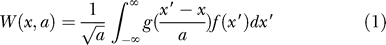


The filter function used in this study was the Morlet wavelet, defined as:


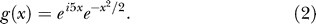


This particular wavelet was chosen because the length scale of the transform corresponds approximately to the period of any localized pattern [36]. The resulting transform values may be plotted in the form of a scalogram (Figure 1B), comprised of a contour plot in which the *x-*axis is the position along the genome *(x),* and the *y*-axis is the length scale *(a)* at which the transform is computed. Given that we employed the Morlet wavelet, this scalogram is useful for elucidating the strength of a range of periodicities localized at each point in time-series data (or, in this case, chromosome position-associated data). The particular voices (i.e., length scales) assessed in the transform for each genome were chosen such that the length scales presented on each scalogram correspond to periods between approximately 1.5% and 20% of the overall genome size.

Currently, no standard statistical methods of verifying patterns identified using continuous wavelet transforms are in common use. Thus, the significance of each transform value was ascertained by a bootstrap approach in which the order of the data points along the chromosome was randomized 200×, and the real and imaginary portions of the Morlet wavelet transform were recomputed for each randomized dataset (described previously for the real portion of the Morlet wavelet [17]). As described in Protocol S1, the randomization of each genome position-associated dataset was performed on either a gene-by-gene basis (for annotation-derived data) or on a kilobase-by-kilobase basis (for annotation-independent properties such as GC content). Thus, the null hypothesis against which each wavelet scalogram was tested consisted of the wavelet transform of a “scrambled” dataset, where the unit of chromosome which was scrambled was either the gene or a kilobase segment. A *p-*value was then computed for each point in the scalogram based upon the number of times the magnitude of the transform value from each randomization exceeded that of the original transform.

The *p-*value cutoff corresponding to a selected FDR [23] (FDR < 5%) was then determined from the distribution of *p-*values computed for each scalogram from the randomization tests. Given this cutoff, one can generate a binary matrix (the same size as the scalogram) containing unity for each point in the scalogram for which FDR < 0.05, and zeroes elsewhere. The ratio of the sum of the non-zero elements in this binary matrix to the total matrix size is taken to be the pattern strength of a given dataset (colored areas in Figure 1B). For matrices of the same size (as for E. coli gene expression, essentiality, and evolutionary retention), the sum of the binary significance matrices yields the degree of pattern overlap, as illustrated schematically in Figure 4A.

### Controls.

Presented in the Protocol S1 are a set of positive and negative controls for the wavelet transform and bootstrap procedure described above. The negative controls showed that no significant patterns were detected in trivial or randomly ordered datasets (for which no pattern would be expected a priori), thus effectively ruling out the possibility that the observed periodic patterns are simply artifacts inherent either in the wavelet filter used or spurious cyclic patterns caused by outliers in otherwise random data (called the Slutzky-Yule effect when observed in moving averages). Wavelet analysis was performed for a 1-Mb subset of the Pseudomonas putida GC/AT dataset in order to rule out the possibility that the correlation shown in Figure 3A was due to an artifact of the wavelet voices chosen for the varying genome sizes. No significant decrease in fractional pattern strength was detected for the smaller subset.

## Supporting Information

Protocol S1 Additional Material Providing More Detailed Experimental Methods and Positive and Negative Controls (195 KB DOC)

Table S1 Complete List of Computed Pattern Strengths for Each Chromosome in this Study, along with Associated Organismal Data (53 KB XLS)

## Abbreviations

CAI: codon adaptation index
FDR: false discovery rate
SD: standard deviation
