# Supplementary material for: Long-Range Periodic Patterns in Microbial Genomes Indicate Significant Multi-Scale Chromosomal Organization
Source: PLoS Comput Biol. 2006 Jan 13;2(1):e2. doi: 10.1371/journal.pcbi.0020002 (PMC1326223; doi:10.1371/journal.pcbi.0020002)
Supplement: Supplementary file 1 [file pcbi.0020002.sd001.doc]

**Long-range periodic patterns in prokaryotic genome sequences indicate significant multi-scale chromosomal organization**

Timothy E. Allen, Nathan D. Price, Andrew R. Joyce, and Bernhard Ø. Palsson

*Supplementary Methods and Controls*

Here we describe positive controls of wavelet analyses of test signals to demonstrate the efficacy of the methods used in this study. Additionally, we show negative controls which demonstrate that the observed patterns are not merely examples of the Slutzky-Yule effect, nor are they artifacts resulting from the wavelet filter function. We also describe the data pre-processing and post-processing at each stage of the analysis.

Positive controls

We generated a test signal of length 4000 (comparable to the length of genome-scale data sets in *E. coli*) consisting of the sum of two sine waves, one having a period of 1/6 the overall length of the data set and the other having a period of 1/12 the overall data length (Fig. S1a). The second test signal consisted of the first with noise added, where the magnitude of the noise varied linearly along the length of the dataset between 0 and 10 times the magnitude of the original signal, with the maximal 10x noise occurring at the midpoint of the data set, or x = 2000 (Fig. S1b).

**Figure S1** Wavelet analysis of test signals. **a.** Sum of two sine waves with wavelengths equal to 1/12 and 1/6 of the overall data length. **b.** Test signal with noise added (linearly uniform random noise between 0 and 10 times the magnitude of the original signal, with maximum noise at center of signal). **c.** Wavelet scalogram (real portion of Morlet wavelet) of original test signal. Insignificant portions of the scalogram from the real and imaginary portions of the Morlet wavelet transform (*FDR* < 5%) are shown in white. **d.** Wavelet scalogram of the noisy test signal shown in (b).

We then performed wavelet analyses of these test signals, as described in the Methods section in the text. The scalogram corresponding to the first test signal clearly showed the two frequencies present, with only slight edge effects (Fig. S1c). The scalogram corresponding to the noisy test signal reveals that the wavelet transform is still able to detect the lower-frequency signal across the length of the data set, even where noise was maximal (at coordinate 2000). Approximately three-fourths of the higher-frequency signal was elucidated and deemed significant (false discovery rate, or *FDR*, < 5%), where significance and FDR are described in the Methods of the main text. The noise in the localized region between points 1500 and 2000 obscured the original high-frequency signal over that span.

Negative controls

Using *FDR* < 5% as our stringency cutoff (see Methods of main text), we then computed the wavelet scalogram and the significant regions of each scalogram for three trivial “data sets”: constant values (ones), uniformly random numbers between 0 and 1, and the *E. coli* gene expression data set for which the locus order has been randomized. These scalograms revealed no significant patterns at all (pattern strength < 1%, as defined in the text).

A potential artifact that may arise when looking for any pattern or period in noisy data sets may be a spurious periodicity that is due to outliers in otherwise random data. (In moving averages of such data, this artifact is called the Slutzky-Yule effect.) For a data set whose dynamic range is fairly high, such as gene expression (for which the dynamic range is nearly 7000), a potential concern would be that the highly-expressed genes would introduce spurious periodicities in either moving average plots or in wavelet analysis scalograms of this data set. To test for this effect, we generated 100 data sets in which the gene expression data were randomized with respect to gene locus. For each of these “shuffled” expression data sets, we then performed the wavelet/randomization test to elucidate regions of significant pattern density (*FDR* < 5%), and we summed binary matrices of these significant regions as determined from the real portion of the Morlet wavelet function to observe any overlap in patterns (as described in the Methods of the main text). As shown in Figure S2, however, the pattern overlap for the randomly-ordered gene expression data sets was minimal, reaching no higher than 18 of the 100 datasets for any point in the scalogram overlap plot. Essentially no significant patterns at all were observed, and certainly not any that even remotely resembled the patterns in any of the actual datasets considered in this study.

**Figure S2** Overlap plot of significant regions of periodicity as determined from the real and imaginary portions of the Morlet wavelet scalogram (*FDR* < 5%) for 100 randomly ordered gene expression data sets. The highest overlap at any given point was 11%, thus indicating that the observed patterns in this study are likely not an artifact resulting from the wavelet approach used.

We also tested whether the pattern strength might be an artifact due to data set length (i.e. accounting for the observation of increasing pattern strength with increasing genome length). Thus, we took short (~500-1000 kb) segments of larger highly pattern genomes (e.g. *Pseudomonas putida*) and ran the wavelet analysis. The original higher-frequency patterns were still detected, thus ruling out a length-dependent artifact inherent in the wavelet method. Additionally, similar patterns to those observed in the data sets using the Morlet wavelet filter function in this study were also observed using another wavelet filter function designed to detect local and global periodic patterns (the Marr wavelet, also known as the “Mexican hat” wavelet). Thus, we are confident that the patterns observed in this study are indeed legitimate and are not simply an artifact resulting from the particular wavelet filter function used or the randomization procedure.

Data pre- and post-processing

Since genes in prokaryotic organisms are not uniformly spaced along the genome and may even overlap slightly, the gene midpoints used as locus values for chromosomal position are not uniform. However, wavelet analysis technically only makes sense when performed on a set of uniform time points (or, in the case of this study, uniformly-spaced chromosomal loci). Thus, for all annotation-dependent datasets examined in this study (including gene expression, essentiality, ORF length, intergenic region length, etc.), it was necessary to linearly pre-interpolate the data before computing the wavelet transform values. This pre-interpolation did not introduce any spurious patterns (or alter any patterns observed in non-uniformly spaced data), probably due to the overall uniformity of ORFs in prokaryotes, as well as the absence of large intergenic regions. There was no need to pre-interpolate any of the annotation-independent data sets (e.g. G/C content, fractional gene density, etc.), as these were already uniform 1-kb segments.

Since some of the data sets (and thus the resulting scalograms) were of varying lengths, they had to be reduced in length to whichever data set contained the least number of elements in order to generate uniformly-sized binary matrices which could be summed for the overlap plots (see Fig. 4a from the main text). Thus, after computing the wavelet transforms (and, for the figures, the moving averages) for each data set, we interpolated each row of the scalogram to the minimum data set length. Since these scalograms (and the resulting *p*-values) were smooth functions because of the nature of the wavelet filter function, we used a piecewise cubic Hermite interpolation to best preserve the observed patterns. Note that this interpolation was *not* performed on the primary data, but only on the wavelet scalograms and moving averages.

Controls for correlation of pattern strength and GC content

The correlation between GC/AT pattern strength and genome length was assessed for high GC content genomes (> 50%, > 55%, > 60%, and > 65%, Figure S3a) and low GC content genomes (< 50%, < 45%, < 40%, and < 35%, Figure S3b). This analysis showed that the strong positive correlation identified and presented in Figure 3 of the manuscript holds for all cases except for the low GC content (<40% and <35% GC), which are presumably the least rigid genomes.

**Figure S3** Correlation of GC/AT pattern strength and genome length for genomes of varying %GC content. Subplot titles specify GC% cutoff values (i.e. the >50% GC plot includes genomes with greater than 50% GC content). Pearson correlation coefficient values are depicted directly on each subplot. **a.** High GC content genome correlations between GC/AT pattern strength and genome length. **b.** Low GC content genome correlations between GC/AT pattern strength and genome length.
